# Revision joint replacement surgeries of the hip and knee across geographic region and socioeconomic status in the western region of Victoria: a cross-sectional multilevel analysis of registry data

**DOI:** 10.1186/s12891-019-2676-z

**Published:** 2019-06-25

**Authors:** Sharon L. Brennan-Olsen, Sara Vogrin, Stephen Graves, Kara L. Holloway-Kew, Richard S. Page, M. Amber Sajjad, Mark A. Kotowicz, Patricia M. Livingston, Mustafa Khasraw, Sharon Hakkennes, Trisha L. Dunning, Susan Brumby, Alasdair G. Sutherland, Jason Talevski, Darci Green, Thu-Lan Kelly, Lana J. Williams, Julie A. Pasco

**Affiliations:** 1 Department of Medicine, The University of Melbourne-Western Health, Level 3, WHCRE Building, Sunshine Hospital, 176 Furlong Road, St Albans, Melbourne, VIC 3021 Australia; 2 Australian Institute for Musculoskeletal Science (AIMSS), The University of Melbourne and Western Health, 176 Furlong Road, St Albans, Melbourne, VIC 3021 Australia; 3 Australian Orthopaedic Association National Joint Replacement Registry, SAHMRI, Level 4, North Terrace, Adelaide, SA 5000 Australia; 4 Deakin University, Pigdon Road, Geelong, VIC 3220 Australia; 5 Barwon Centre for Orthopaedic Research and Education (B-CORE), Barwon Health and St John of God Hospital, Myers Street, Geelong, VIC 3220 Australia; 6 Barwon Health, University Hospital Geelong, Ryrie Street, Geelong, VIC 3220 Australia; 7 University of Sydney, Parramatta Road, Camperdown, Sydney, NSW 2006 Australia; 8 National Centre for Farmer Health, Western District Health Service, Tyers Street, Hamilton, VIC 3300 Australia; 9 South West Healthcare, 25 Ryot Street, Warrnambool, VIC 3280 Australia; 10 School of Pharmacy and Medical Sciences, University of South Australia, City East Campus, North Terrace, Adelaide, SA 5001 Australia; 11 Department of Preventive Medicine and Epidemiology, Monash University, Alfred Centre, 99 Commercial Road, Prahran, VIC 3004 Australia

**Keywords:** Epidemiology, Geographic region, Revision joint replacements, Registry data, Social disadvantage

## Abstract

**Background:**

Residents of rural and regional areas, compared to those in urban regions, are more likely to experience geographical difficulties in accessing healthcare, particularly specialist services. We investigated associations between region of residence, socioeconomic status (SES) and utilisation of all-cause revision hip replacement or revision knee replacement surgeries.

**Methods:**

Conducted in western Victoria, Australia, as part of the Ageing, Chronic Disease and Injury study, data from the Australian Orthopaedic Association National Joint Replacement Registry (2011–2013) for adults who underwent a revision hip replacement (*n* = 542; 54% female) or revision knee replacement (*n* = 353; 54% female) were extracted. We cross-matched residential addresses with 2011 census data from the Australian Bureau of Statistics (ABS), and using an ABS-derived composite index, classified region of residence according to local government areas (LGAs), and area-level SES into quintiles. For analyses, the control population (*n* = 591,265; 51% female) was ABS-determined and excluded adults already identified as cases. Mixed-effects logistic regression was performed.

**Results:**

We observed that 77% of revision hip surgeries and 83% of revision knee surgeries were performed for residents in the three most socially disadvantaged quintiles. In adjusted multilevel models, total variances contributed by the variance in LGAs for revisions of the hip or knee joint were only 1% (SD random effects ±0.01) and 3% (SD ± 0.02), respectively. No differences across SES or sex were observed.

**Conclusions:**

No differences in utilisation were identified between SES groups in the provision of revision surgeries of the hip or knee, independent of small between-LGA differences.

## Mini abstract

Residents of rural/regional areas experience more difficulty accessing specialist healthcare providers compared to urban residents. Socially advantaged groups had the greatest uptake of arthroplasty, independent of small between-area differences. Despite few differences in revision surgery uptake across social groups, we caution against assumptions of no differences in need.

## Background

The risk for joint diseases, such as arthritis, is greater in rural and farming communities when compared to the general population, due to occupational exposures related primarily to the agricultural industry [1–3]. Rural and regional residents experience out-of-pocket costs when seeking healthcare, and the inequities in specialist health care are enhanced by the effects of these costs [3, 4]. As we have previously suggested, and compared to urban residents, the uptake of elective surgery such as joint revision for rural/regional populations may be lower than the expected need for the revision surgery [1–3], an association often suggested as being related to geographical distance between patient and health service provider [3, 5, 6].

A recent study by McGrory et al. examined current hip and knee revision surgery burden across Australia [7]. Until recently, the pattern of primary total joint replacement and relationships with age, sex, geographic location and socioeconomic status (SES) had not been described for the region of western Victoria [8]. One of the most important outcome measures of joint replacement surgery is revision rate [9]. We aimed to describe the pattern of revision surgeries as part of the Ageing, Chronic Disease and Injury (ACDI) study, which was launched to contribute locally-generated knowledge regarding chronic disease in this region [10].

## Methods

### Australian Orthopaedic Association national joint replacement registry

The Australian Orthopaedic Association National Joint Replacement Registry (AOANJRR) was established to monitor joint replacements from all public and private hospitals Australia-wide [11], and is the most complete and extensive set of joint replacement data in Australia [11]. As previously reported, AOANJRR data are cross-referenced with government hospital separation data as a verification process [12].

In our current analyses, we investigated revision joint replacement surgeries (performed for any diagnosis), whereby a revision joint replacement surgery was defined as “…re-operations of previous [joint] replacements where one or more of the prosthetic components are replaced, removed, or one or more components are added” [13], inclusive of all types of implants. We extracted data pertaining to the 941 revision joint replacement surgeries that had been performed during 2011–2013, which encompassed knee or hip (*n* = 895); shoulder (*n* = 28); and elbow, ankle or wrist (*n* = 18). Due to cell counts, we examined only revision joint replacements of the knee and hip.

### Study population: cases, controls, and socioeconomic position

Cases were defined as adults residing in western Victoria who had undergone a revision joint replacement of the knee (*n* = 4179; 56% female) and/or hip (*n* = 3120; 54% female). We matched each patient’s residential address to the Australian Bureau of Statistics (ABS) 2011 census data and, using the ABS-derived Index of Relative Socioeconomic Advantage and Disadvantage (IRSAD), defined area-level SES into quintiles based on cut-points for the Victorian population.

From the ABS cross-matching process, and using ABS concordance files, we identified the Local Government Area (LGA) within which cases and controls resided: of which the control population was 591,265 (51% female). As previously published, we assumed that population figures remained similar between 2011 and 2013 [8].

### Statistical analyses

We used similar multilevel modelling procedures (mixed effects logistic regression) employed in our previous studies [8] to now investigate the effect of various social factors on the revision of a knee or hip joint replacement. Analyses were performed using Stata 13.0 (StataCorp, Release 13, LP, College Station, Texas, USA).

## Results

Table 1 presents descriptive characteristics of patients registered with the AOANJRR (2011–13) as having undergone a revision joint replacement surgery of the hip (*n* = 542) or knee (*n* = 353). We report that the greatest proportions of revision joint replacements of the hip and knee, respectively, were observed in women (53.7 and 54.1%), those aged 60–69 years (31.5 and 36.8%) and 70–79 years (32.5 and 30.3%), and in the three most socially disadvantaged quintiles. The three most common reasons for revision of the hip joint were loosening/lysis (39.7%), metal related pathology (15.1%), and infection (13.6%). For revision of the knee joint, the three most common reasons were loosening/lysis (37.1%), infection (21.8%), and pain (11.6%).

**Table 1:** Characteristics of residents from Western Victoria who underwent a hip or knee revision surgery, 2011–13

	Revision joint replacement surgeries		
	Total (*n* = 895)	Hip (*n* = 542)	Knee (*n* = 353)
Women, n (%)	482 (53.8%)	291 (53.7%)	191 (54.1%)
Age group (years), n (%)			
0–49	37 (4.1%)	21 (3.9%)	16 (4.5%)
50–59	115 (12.8%)	66 (12.2%)	49 (13.9%)
60–69	301 (33.6%)	171 (31.5%)	130 (36.8%)
70–79	283 (31.6%)	176 (32.5%)	107 (30.3%)
≥ 80	159 (17.8%)	108 (19.9%)	51 (14.4%)
Socioeconomic quintiles, n (%)			
Quintile 1^b^	236 (26.4%)	140 (25.8%)	96 (27.2%)
Quintile 2	258 (28.8%)	139 (25.6%)	119 (33.7%)
Quintile 3	219 (24.5%)	140 (25.8%)	79 (22.4%)
Quintile 4	148 (16.5%)	99 (18.3%)	49 (13.9%)
Quintile 5^c^	34 (3.8%)	24 (4.4%)	10 (2.8%)
^a^Reasons for joint replacement, n (%)			
Arthrofibrosis	10 (1.1%)	–	10 (2.8%)
Erosion ^d^	11 (1.2%)	5 (0.9%)	6 (1.7%)
Fracture	49 (5.5%)	46 (8.5%)	3 (0.8%)
Implant breakage ^e^	20 (0.6%)	13 (2.4%)	7 (2.0%)
Infection	151 (16.9%)	74 (13.6%)	77 (21.8%)
Instability	20 (2.2%)	1 (0.2%)	19 (5.4%)
Loosening/lysis	346 (38.7%)	215 (39.7%)	131 (37.1%)
Metal-related pathology	95 (10.6%)	82 (15.1%)	13 (3.7%)
Other ^f^	37 (4.1%)	24 (4.4%)	13 (3.7%)
Pain	54 (6.0%)	13 (2.4%)	41 (11.6%)
Progression of disease	21 (2.3%)	–	21 (5.9%)
Prosthesis dislocation	65 (7.3%)	63 (11.6%)	2 (0.6%)
Wear ^g^	16 (1.8%)	6 (1.1%)	10 (2.8%)

^a^Revisions may have been performed for more than one reason

^b^ Most disadvantaged; ^c^ Most advantaged; ^d^ erosion includes chrondrolyses, acetabular, patella erosion; ^e^ implant breakage of the acetabular, femoral, head, patella, tibial, tibial insert, stem; ^f^ other includes bearing dislocation, heterotopic bone, leg length discrepancy, malalignment, malposition, osteonecrosis, synovitis, tumour; ^g^ ‘wear’ of the acetabular insert, patella, tibial, tibial insert

Results from the multilevel modelling are presented in Table 2. The likelihood of revisions of the hip or knee differed minimally across the LGAs; differences were 2% (SD of random effects ±0.01) and 5% (SD ± 0.03), respectively. In fully adjusted multilevel models, these differences were reduced, whereby the total variance in revisions of the hip or knee contributed by the variance of LGAs was 1.0% (SD of random effects ±0.01), and 3.0% (SD ± 0.02), respectively. In fully adjusted multilevel models, no sex differences were observed for revisions of the hip or knee.

**Table 2:** Multilevel logistic regression models showing effects of sex, age and socioeconomic status on revision surgery

	Hip revisions *Odds ratios (95%CI)*	*p value*	Knee revisions *Odds ratios (95%CI)*	*p value*
Women	1.01 (0.85–1.19)	0.94	1.04 (0.84–1.29)	0.69
Men (referent)	1.00	–	1.00	–
Age group (years)				
0–39	**0.01 (0.003–0.02)**	**≤0.001**	**0.01 (0.004–0.02)**	**≤0.001**
40–49	**0.08 (0.04–0.13)**	**≤0.001**	**0.07 (0.04–0.13)**	**≤0.001**
50–59	**0.32 (0.24–0.42)**	**≤0.001**	**0.31 (0.23–0.44)**	**≤0.001**
60–69 (referent)	1.00	–	1.00	–
70–79	**1.68 (1.36–2.07)**	**≤0.001**	**1.32 (1.02–1.71)**	**0.03**
≥ 80	**1.49 (1.17–1.90)**	**0.001**	0.89 (0.64–1.23)	0.47
Socioeconomic status				
Quintile 1^a^	0.85 (0.67–1.09)	0.20	1.11 (0.81–1.51)	0.52
Quintile 2	0.87 (0.68–1.10)	0.25	**1.37 (1.02–1.83)**	**0.03**
Quintile 3 (referent)	1.00	–	1.00	–
Quintile 4	1.11 (0.85–1.44)	0.44	0.98 (0.68–1.40)	0.92
Quintile 5^b^	0.86 (0.56–1.34)	0.51	0.65 (0.34–1.27)	0.21
Random effects of LGAs^c^	0.20 (0.11–0.36)		0.33 (0.17–0.63)	
% total variance contributed by LGAs^d^	1%		3%	
*p* value^e^		**≤0.001**		**≤0.001**

^a^Most disadvantaged quintile of socioeconomic status; ^b^Most advantaged quintile of socioeconomic status; ^c^Standard deviation of the random effects of local government areas (LGAs); ^d^Percentage of the total variance contributed by the variance of the LGAs; ^e^*p* value for a likelihood ratio test. Bold text indicates significance at *p* value ≤0.05

## Discussion

In the ACDI study region of western Victoria, Australia, we observed the greatest proportions of revision joint replacements of the hip and knee, respectively, in women, and in those aged 60 years or older. Approximately 80% of revision surgeries at the hip and knee were utilised by residents in the three most socially disadvantaged quintiles. However, after adjustments, multilevel modelling showed that total variances in revisions of the hip or knee joint contributed by the variance in LGAs were minor, and no differences between SES groups were observed.

The lack of differences in revision surgeries between SES groups contrasts with the expected higher rates of need for revisions in socially disadvantaged populations. We may speculate as to the lack of differences in the uptake of revision joint replacements across SES. It is possible that, as we have previously suggested for primary joint replacements [8], socially disadvantaged individuals may delay the utilisation of revision surgeries for a longer period than socially advantaged individuals. Delayed utilisation of elective surgeries by disadvantaged individuals may be related to a limited capacity to cover out-of-pocket costs, the lack of social support during recovery, or the inability to take leave from employment. It may also be possible that the joint replacements of socially disadvantaged individuals experience greater ‘wear and tear’ than socially advantaged individuals due to having more physically demanding occupations [14]. The lack of associations we observed may be explained by the opposing factors of advantaged individuals undergoing revision surgeries without postponement, and disadvantaged individuals experiencing greater ‘wear and tear’ and thus having increased need.

Differences in preventive lifestyle behaviours may also result in variation in the need for surgeries. For instance, a randomised trial of exercise therapy vs patient education in 109 patients with hip osteoarthritis showed that exercise therapy reduced the need for primary joint replacement by 44% [15]. While there are no data investigating the role of exercise therapy on the need for revision joint replacement, exercise has been shown as beneficial in reducing pain and improving function in those with osteoarthritis, and thus it may be plausible that exercise therapy may result in reduced need for revision surgeries. The association between social advantage and physical activity has been well-documented [16, 17], and, although general exercise per se is not targeted at improving joint health, nonetheless, when contrasted with possible increased need yet delayed provision of revision surgeries by disadvantaged individuals, this may provide another plausible explanation as to why we did not observe any differences between social groups.

We observed similar rates of revision joint replacement surgeries for men and women. It is well-established that the prevalence of end-stage knee and/or hip arthritis, a condition that generally requires joint replacement surgery, is higher among women than men. Studies have shown that women with arthritis of the hip or knee suffer worse symptoms and greater disability, but may be less likely to undergo joint replacement surgery [18]. Should this be the case with revision surgeries, this might contribute to the lack of between-sex differences observed in the ACDI region.

Our study contributes to the emerging evidence-base regarding the ACDI region in terms of musculoskeletal disease, particularly revision joint replacements. Moreover, the findings are founded on the comprehensive data from the AOANJRR. We note limitations with this study, which should be considered when interpreting our findings. First, we may have been limited in our sample size to identify differences between the SES groups. Our analyses included all-cause revisions of the knee and hip, which encompassed a relatively small geographic area. Due to small numbers (*n* = 28) we were unable to investigate joint revisions of the shoulder. Obesity is likely to be a confounder in revision joint replacements and may possibly explain the greater proportion of revision surgeries in patients in the lower three quartiles of SES; however, this information, and other potentially confounding variable data, are not collected as part of registry data. The denominator for these analyses was everyone in the BSD region, rather than those that had a primary joint replacement: data linkage between primary and revision joint replacement over only a 3 year period would yield small numbers and was beyond the scope of this current investigation that was focused on informing the larger ACDI study. SES may have an impact on time to revision, rather than revision overall. Our analyses of LGAs and SES makes assumptions regarding heterogeneity of those residing in those areas, and finally, we acknowledge that the uptake of revision surgeries does not equate to need for surgery, nor does it reflect disease state.

## Conclusions

In conclusion, although small between-LGA differences in utilisation were observed, no differences were detected between SES groups in the provision of revision TKR and THR. We speculate as to reasons for a lack of differences in revision surgeries across social groups, but caution against assumptions that no difference in need or uptake exists.

## Data Availability

The datasets generated and analysed during the current study are not publicly available due to the information collected by the AOANJRR being protected by quality assurance confidentiality (under the Health Insurance Act of 1973), which ensures that patients, surgeons, hospitals and government information supplied to the AOANJRR remains confidential and secure. The AOANJRR governs the dataset supporting the conclusions of this article (https://aoanjrr.sahmri.com/).

## Abbreviations

ABS: Australian Bureau of Statistics
ACDI: Ageing, Chronic Disease and Injury (study)
AOANJRR: Australian Orthopaedic Association National Joint Replacement Registry
AOR: Adjusted odds ratio
BSD: Barwon Statistical Division
IRSAD: Index of Relative Socioeconomic Advantage and Disadvantage
LGA: Local Government Area
OR: Odds ratio
SD: Standard deviation
SES: Socioeconomic status
